# Supplementary material for: Diagnosis and Prognosis of COVID-19 Disease Using Routine Blood Values and LogNNet Neural Network
Source: Sensors (Basel). 2022 Jun 25;22(13):4820. doi: 10.3390/s22134820 (PMC9269123; doi:10.3390/s22134820)
Supplement: Supplementary file 1 [file sensors-22-04820-s001.zip › SARS-CoV-2-RBV(1-2)/Readme.pdf]

Instruction and Grant of License for SARS-CoV-2-RBV(1-2) data set

In the EBYU-MG hospital, only the cases that were detected as SARS-CoV-2 by real-time reverse transcriptase polymerase chain reaction (RT-PCR) in nasopharyngeal or oropharyngeal swabs during the dates covered by this study were diagnosed with COVID-19. The research only included individuals over the age of 18. In order to prevent various complications, only RBV results at the first admission were recorded. In our data set, there are no individual characteristics (name-surname, identification number, etc.) that define the patients.

As a matter of fact, it has been stated in previous studies that this pathogen can cause a serious respiratory disorder that requires special intervention in intensive care units (ICUs).

First SARS-CoV-2-RBV data set (SARS-CoV-2-RBV1) includes the information of 2648 patients diagnosed with COVID-19 and receiving outpatient treatment in hospital on the specified dates, and the same number of patients (control group) whose COVID-19 tests were negative.

The dataset contains 2 files:

- File 1: SARS-CoV-2-RBV1.txt

First column: Diagnosis.

In the SARS-CoV-2-RBV1 data set, positive COVID-19 test results were coded as 1 and negative as 0 (COVID-19 = 1, non-COVID-19 = 0).

Other columns futures are presented in Table 1. SARS-CoV-2-RBV1 dataset include immunological, hematological and biochemical RBV parameters and consists of 51 features.

- File 2: SARS-CoV-2-RBV1.sav
  IBM SPSS Statistics format

Second SARS-CoV-2-RBV data set (SARS-CoV-2-RBV2) includes the information of 3899 patients who were treated for COVID-19 in hospital on the specified dates. The treatment units of these patients at the first admission were examined. In the SARS-CoV-2-RBV2 dataset, there were $n = 203$ ICU and $n = 3696$ non-ICU COVID-19 patients.

The dataset contains 2 files:

- File 1: SARS-CoV-2-RBV2.txt
  First column: Treatment Units/Service (ICU or non-ICU).
  In the SARS-CoV-2-RBV2 dataset, severely infected (ICU) COVID-19 patients were coded as 1, while mildly infected (non-ICU) COVID-19 patients were coded as 0.
  Other columns futures are presented in Table 2.
  SARS-CoV-2-RBV2 dataset include immunological, hematological and biochemical RBV parameters and consists of 51 features.

- File 2: SARS-CoV-2-RBV2.sav
  IBM SPSS Statistics format

Grant of License

We grant You a non-exclusive, non-transferable, revocable license to use the **SARS-CoV-2-RBV** Dataset solely for Your non-commercial, educational, and research purposes only, but without any right to copy or reproduce, publish or otherwise make available to the public or communicate to the public, sell, rent or lend the whole or any constituent part of the **SARS-CoV-2-RBV** Dataset thereof.

Kindly cite our paper when you wish to use this dataset:

Huyut, M.T.; Velichko, A. Diagnosis and Prognosis of COVID-19 Disease Using Routine Blood Values and LogNNet Neural Network. *Sensors* **2022**, *22*

Table 1. Feature numbering for SARS-CoV-2-RBV1 datasets.

№	Feature	№	Feature	№	Feature	№	Feature	№	Feature
1	CRP	12	NEU	23	MPV	34	GGT	45	Sodium
2	D-Dimer	13	PLT	24	PDW	35	Glucose	46	T-Bil
3	Ferritin	14	WBC	25	RBC	36	HDL-C	47	TP
4	Fibrinogen	15	BASO	26	RDW	37	Calcium	48	Triglyceride
5	INR	16	EOS	27	ALT	38	Chlorine	49	eGFR
6	PT	17	HCT	28	AST	39	Cholesterol	50	Urea
7	PCT	18	HGB	29	Albumin	40	Creatinine	51	UA
8	ESR	19	MCH	30	ALP	41	CK		
9	Troponin	20	MCHC	31	Amylase	42	LDH		
10	aPTT	21	MCV	32	CK-MB	43	LDL		
11	LYM	22	MONO	33	D-Bil	44	Potassium		

CRP: C-reactive protein; INR: international normalized ratio; PT: prothrombin time; PCT: Procalcitonin; ESR: erythrocyte sedimentation rate; aPTT: activated partial prothrombin time; LYM: lymphocyte count; NEU: neutrophil count; PLT: platelet count; WBC: white blood cell count; BASO: basophil count; EOS: eosinophil count; HCT: hematocrit; HGB: hemoglobin; MCH: mean corpuscular hemoglobin; MCHC: mean corpuscular hemoglobin concentration; MCV: mean corpuscular volume; MONO: monocyte count; MPV: mean platelet volume; PDW: platelet distribution width; RBC: red blood cells; RDW: red cell distribution width; ALT: alanine aminotransaminase; AST: aspartate aminotransferase; ALP: alkaline phosphatase; CK-MB: creatine kinase myocardial band; D-Bil: direct bilirubin; GGT: gamma-glutamyl transferase; HDL-C: high-density lipoprotein-cholesterol; CK: creatine kinase; LDH: lactate dehydrogenase; LDL: low-density lipoprotein; T-Bil: total bilirubin; TP: total protein; eGFR: estimating glomerular filtration rate; UA: uric acid.

Table 2. Feature numbering for SARS-CoV-2-RBV2 datasets.

№	Feature	№	Feature	№	Feature	№	Feature	№	Feature
1	ALT	12	Chlorine	23	eGFR	34	MONO	45	Fibrinogen
2	AST	13	Cholesterol	24	Urea	35	MPV	46	INR
3	Albumin	14	Creatinine	25	UA	36	NEU	47	PT
4	ALP	15	CK	26	BASO	37	PDW	48	PCT
5	Amylase	16	LDH	27	EOS	38	PLT	49	ESR
6	CK-MB	17	LDL	28	HCT	39	RBC	50	Troponin
7	D-Bil	18	Potassium	29	HGB	40	RDW	51	aPTT
8	GGT	19	Sodium	30	LYM	41	WBC		
9	Glucose	20	T-Bil	31	MCH	42	CRP		
10	HDL-C	21	TP	32	MCHC	43	D-Dimer		
11	Calcium	22	Triglyceride	33	MCV	44	Ferritin		

ALT: alanine aminotransaminase; AST: aspartate aminotransferase; ALP: alkaline phosphatase; CK-MB: creatine kinase myocardial band; D-Bil: direct bilirubin; GGT: gamma-glutamyl transferase; HDL-C: high-density lipoprotein-cholesterol; CK: creatine kinase; LDH: lactate dehydrogenase; LDL: low-density lipoprotein; T-Bil: total bilirubin; TP: total protein; eGFR: estimating glomerular filtration rate; UA: uric acid; BASO: basophil count; EOS: eosinophil count; HCT: hematocrit; HGB: hemoglobin; LYM: lymphocyte count; MCH: mean corpuscular hemoglobin; MCHC: mean corpuscular hemoglobin concentration; MCV: mean corpuscular volume; MONO: monocyte count; MPV: mean platelet volume; NEU: neutrophil count; PDW: platelet distribution width; PLT: platelet count; RBC: red blood cells; RDW: red cell distribution width; WBC: white blood cell count; CRP: C-reactive protein; INR: international normalized ratio; PT: prothrombin time; PCT: procalcitonin; ESR: erythrocyte sedimentation rate; aPTT: activated partial prothrombin time.
